# *Staphylococcus aureus* SspA (V8 protease): New skin pathogenesis insights into an old enzyme

**DOI:** 10.1371/journal.ppat.1013048

**Published:** 2025-04-24

**Authors:** John C. Thorstenson, Alexander R. Horswill

**Affiliations:** 1 Department of Immunology & Microbiology, School of Medicine, University of Colorado Anschutz Medical Campus, Aurora, Colorado, United States of America; 2 Department of Veterans Affairs, Eastern Colorado Health Care System, Aurora, Colorado, United States of America; University of Geneva: Universite de Geneve, SWITZERLAND

## Proteases of bacterial pathogens and the discovery of *Staphylococcus aureus* SspA (V8 protease)

Proteases are ubiquitous enzymes that catalyze peptide bond hydrolysis, and bacterial pathogens utilize proteases to facilitate their invasion and persistence in host environments. For example, *Streptococcus pyogenes* uses SpeB to degrade fibrinogen and other extracellular matrix proteins for tissue invasion, and *Yersinia pestis* triggers macrophage cell death by inactivating MAPK and NF-κB signaling via YopJ [1,2]. *Staphylococcus aureus*, a versatile human pathogen, is famous for its vast array of virulence factors that enable infection of nearly every body site. Most *S. aureus* strains encode 10 extracellular proteases with unique substrate specificities and mechanisms that contribute to pathogenesis.

The first *S. aureus* extracellular protease was identified more than 50 years ago and was isolated from strain V8 by Drapeau and colleagues [3]. V8 protease (SspA) is a serine protease with high specificity for peptide bond hydrolysis on the carboxy side of glutamate residues, though it has been shown to also be capable of cleaving after aspartate residues to a lesser extent. Given its unique substrate specificity and ease to natively purify in large quantities, SspA is extensively used commercially to digest proteins for peptide analysis by mass spectrometry across many disciplines.

The *sspABC* locus encodes V8 protease (*sspA*), cysteine protease staphopain B (*sspB*), and a small protein inhibitor of staphopain B (*sspC*) [4]. Although *S. aureus* protease regulation is quite nuanced, the *ssp* operon is positively regulated by the Agr quorum sensing system and inhibited by SarA [5]. SspA is secreted as a 36.3 kDa zymogen with an N-terminal propeptide that is proteolytically removed via intermolecular proteolysis and a final cleavage by an additional *S. aureus* protease, aureolysin (Aur), which releases the 29.1 kDa enzymatically active SspA [6]. The first high-resolution crystallographic structure of SspA (PDB ID code 1QY6) revealed that this enzyme folds similarly to several other serine proteases, such as trypsin and the staphylococcal epidermolytic toxins A and B [7]. While highly homologous glutamyl endopeptidases are common amongst other Gram-positive opportunistic pathogens, such as *Staphylococcus epidermidis* (Esp) and *Enterococcus faecalis* (SprE), a unique feature that distinguishes SspA from these other proteases is the presence of a 52-residue C-terminal domain (CTD) of unknown function [8,9]. The SspA CTD is highly conserved amongst *S. aureus* and contains a repeating P-[DN]-N motif, which suggests propensity for type II polyproline helix (PPII) formation. PPII helices are highly flexible secondary structures that often serve as modules for biologically relevant protein–protein interactions, though this has not been demonstrated for the SspA CTD [10,11]. Despite the discovery of SspA over 50 years ago, its specific roles in *S. aureus* pathogenesis have only recently begun to be understood.

## SspA contributes to *Staphylococcus aureus* virulence and pathogenesis on the skin

*S. aureus* is the leading cause of skin and soft-tissue infections globally, which can progress to fatal invasive diseases if left untreated. Additionally, while *S. aureus* colonization of healthy skin is often transient, over 70% of lesions in patients with atopic dermatitis, a chronic skin disease, are persistently colonized by *S. aureus* which may directly exacerbate symptoms [12]. Thus, understanding the molecular mechanisms driving *S. aureus* skin colonization and infection may lead to development of novel therapeutics for *S. aureus* infections and chronic skin diseases.

Generally, *S. aureus* proteases contribute to pathogenesis through tissue degradation, immune evasion, nutrient acquisition, biofilm formation, and limiting virulence factor output. A well-known example of *S. aureus* proteases causing skin pathology is staphylococcal scalded skin syndrome. *S. aureus* epidermolytic toxins A and B (ETA, ETB), which are both glutamyl endopeptidases like SspA, cleave desmoglein-1 in the upper epidermis, causing epidermal detachment and blistering [13]. However, more recently evolved *S. aureus* strains, such as members of the USA300 lineage, do not encode or express these proteases. Despite this, *S. aureus* infections still significantly disrupt epidermal barrier integrity through proteases like SspA.

**Fig 1:**
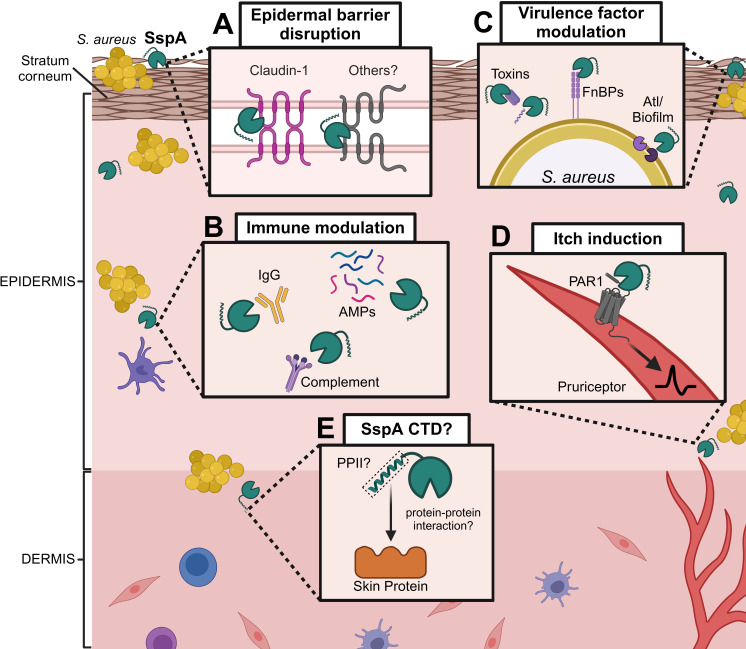
Mechanisms of *Staphylococcus aureus* SspA-mediated virulence in the skin. *S. aureus* SspA serves several identified functions during skin infection, including disruption of the epidermal barrier in the upper epidermis, potentially through tight junction protein degradation **(A)**; degradation of immune factors in the lower epidermis/upper dermis **(B)**; modification of its own secreted and surface-associated virulence factors **(C)**; and induction of itch via PAR1 activation in the lower epidermis **(D)**. The SspA C-terminal domain (CTD) may contribute to virulence, possibly by mediating protein-protein interactions with skin components **(E)**. FnBPs, fibronectin-binding proteins; Atl, autolysin; IgG, immunoglobulin G; AMPs, antimicrobial peptides; PAR1, protease-activated receptor 1; CTD, C-terminal domain; PPII, type II polyproline helix. Created with BioRender.

*S. aureus* cell-free supernatants damage keratinocyte barrier integrity in a *sspA*-dependent manner. Furthermore, recombinant SspA is sufficient to induce this barrier damage in a dose-dependent manner, which has been proposed to proceed through the degradation of tight junction (TJ) proteins such as claudin-1 (Fig 1A) [14,15]. In a murine model, direct application of SspA to the skin led to epidermal barrier dysfunction, as measured by trans-epidermal water loss and riboflavin penetration into the stratum corneum—the outermost layer of the epidermis [16]. While these studies indicate that SspA drives *S. aureus*-induced epidermal barrier dysfunction, the primary SspA substrates leading to this damage *in vivo* remain largely unknown. Despite the ability of SspA to degrade claudin-1 *in vitro* [14] and the observation that *S. aureus* proteases disrupt spatial organization of other TJ proteins occludin and ZO-1 [15], these mechanisms have not been experimentally demonstrated to be the cause of SspA-induced barrier disruption *in vivo*.

In addition to directly interfering with skin barrier integrity, SspA mediates *S. aureus* immune evasion through the degradation of host-derived immune components, which may modulate the skin immune environment. SspA degrades most immunoglobulin classes, including IgG, IgM, and IgA [17]. Following systemic infection with *S. aureus*, the host also mounts a B-cell response resulting in production of SspA-targeting neutralizing antibodies, and individuals persistently colonized with *S. aureus* harbor SspA-specific antibodies, underscoring the importance of SspA in *S. aureus* pathogenesis [18,19]. SspA also inhibits the human complement system through the efficient degradation of C1q, C3, C5, and others [20]. Additional targets that could mediate bacterial immune evasion and dissemination include members of the clotting cascade, host protease inhibitors, and antimicrobial peptides (Fig 1B) [21]. However, the consequences of degradation of these immune factors to *S. aureus* success in skin colonization and infection have not yet been fully evaluated.

Beyond promoting degradation of host factors, *S. aureus* proteases also modulate levels of its own virulence factors that contribute to pathogenesis. A specific example for SspA is its ability to degrade the fibronectin-binding proteins, which are important surface adhesins that mediate attachment of *S. aureus* to host cells [22]. Further, *sspA* is required for biofilm formation in *S. aureus* strain Newman, which is proposed to proceed through proteolytic modulation of autolysin activity, and biofilm formation is an important process in *S. aureus* pathogenesis on the skin and in other models of infection [9,23].

Recently, a highly specific role for SspA during *S. aureus* skin infection was identified. Deng and colleagues found that in a murine epicutaneous infection model, *S. aureus* induced a strong itching response, which led to a significant amount of scratch-induced skin damage [24]. Through systematically testing *S. aureus* mutants, *sspA* was found to be required for itch. SspA is also sufficient to induce itching; intradermal injection of pure SspA elicited a strong itch response comparable to the known itch-inducing agent histamine. The authors also identified an important host target involved in this process—protease-activated receptor 1 (PAR1). SspA activates PAR1 expressed in pruriceptor sensory neurons, which leads to neuronal activation and transmission of the sensation to itch (Fig 1D). This phenomenon proceeds independent of immune-mediated itch, revealing the direct ability of *S. aureus* to induce itch in an animal model [24].

While this mechanism of *S. aureus*-induced itch has been well characterized, numerous outstanding questions remain from the bacterial perspective. *sspA* is upregulated during skin colonization, but the skin-specific signals and bacterial sensory systems contributing to this transcriptional change remain elusive. While the *agr* system is required, there may be additional regulatory mechanisms driving increased SspA production on the skin. Additionally, the SspA-activating enzyme, Aur, is not required for itch, which suggests the presence of an alternative protease native to the skin environment that may activate SspA [24]. Finally, it has not been tested whether the SspA CTD is required for SspA proteolytic activity, and it is possible that SspA CTD PPII helix formation could potentially facilitate strong protein–protein interactions with host targets in the skin, such as PAR1, and contribute to itch induction or epidermal barrier disruption (Fig 1E). Altogether, this study has unveiled a novel, specific virulence function for SspA during skin infection and has revived interest in this old enzyme.

## Summary

Although *S. aureus* SspA was first isolated over 50 years ago, we are only beginning to understand its role in bacterial pathogenesis, especially during skin colonization and infection. SspA contributes to *S. aureus* virulence on the skin by disrupting the epithelial barrier, modulating the immune environment, and manipulating the host sensory nervous system to drive itch, causing the host to further disrupt the skin barrier through scratching. While the primary host target driving itch has been identified, much work remains to fully elucidate the primary SspA substrates in the skin that *S. aureus* degrades to permit skin colonization and infection.
